# Regenerative Medicine and Diabetes: Targeting the Extracellular Matrix Beyond the Stem Cell Approach and Encapsulation Technology

**DOI:** 10.3389/fendo.2018.00445

**Published:** 2018-08-31

**Authors:** Andrea Peloso, Antonio Citro, Tamara Zoro, Lorenzo Cobianchi, Arianna Kahler-Quesada, Carlo M. Bianchi, Axel Andres, Ekaterine Berishvili, Lorenzo Piemonti, Thierry Berney, Christian Toso, Graziano Oldani

**Affiliations:** ^1^Division of Abdominal Surgery, Department of Surgery, Faculty of Medicine, University of Geneva, Geneva, Switzerland; ^2^HepatoPancreato-Biliary Centre, Geneva University Hospitals, Geneva, Switzerland; ^3^San Raffaele Diabetes Research Institute, IRCCS San Raffaele Scientific Institute, Milan, Italy; ^4^Department of General Surgery, IRCCS Policlinico San Matteo, Pavia, Italy; ^5^Department of Clinical, Surgical, Diagnostic and Paediatric Sciences, University of Pavia, Pavia, Italy; ^6^Cell Isolation and Transplantation Center, University of Geneva, Geneva, Switzerland; ^7^Institute of Medical Research, Ilia State University, Tbilisi, Georgia

**Keywords:** pancreas bioengineering, extracellular matrix, stem cells, decellularization, regenerative medicine, organ bioengineering

## Abstract

According to the Juvenile Diabetes Research Foundation (JDRF), almost 1. 25 million people in the United States (US) have type 1 diabetes, which makes them dependent on insulin injections. Nationwide, type 2 diabetes rates have nearly doubled in the past 20 years resulting in more than 29 million American adults with diabetes and another 86 million in a pre-diabetic state. The International Diabetes Ferderation (IDF) has estimated that there will be almost 650 million adult diabetic patients worldwide at the end of the next 20 years (excluding patients over the age of 80). At this time, pancreas transplantation is the only available cure for selected patients, but it is offered only to a small percentage of them due to organ shortage and the risks linked to immunosuppressive regimes. Currently, exogenous insulin therapy is still considered to be the gold standard when managing diabetes, though stem cell biology is recognized as one of the most promising strategies for restoring endocrine pancreatic function. However, many issues remain to be solved, and there are currently no recognized treatments for diabetes based on stem cells. In addition to stem cell resesarch, several β-cell substitutive therapies have been explored in the recent era, including the use of acellular extracellular matrix scaffolding as a template for cellular seeding, thus providing an empty template to be repopulated with β-cells. Although this bioengineering approach still has to overcome important hurdles in regards to clinical application (including the origin of insulin producing cells as well as immune-related limitations), it could theoretically provide an inexhaustible source of bio-engineered pancreases.

## Introduction

Diabetes is a syndrome characterized by an absolute or relative β-cell deficiency in terms of mass (Type 1 diabetes mellitus, T1DM) or function (Type 2 diabetes mellitus, T2DM). Both of these conditions result in an impaired glucose homeostasis.

Diabetes has reached pandemic levels, afflicting over 300 million people worldwide (1) with a cost of care estimated around $176 billion/year in the United States alone (2). Furthermore, the costs resulting from chronic diabetes-related complications like cardio-vascular disease, nephropathy and retinopathy, are growing exponentially (2, 3).

The present standard cure for treating patients with T1DM consists of daily exogenous insulin injections, whereas physical exercise, specific diet, and oral hypoglycemic treatment are the first line of treatments for T2DM. However, exogenous insulin remains a suboptimal treatment, and is far from reaching an adequate regulation of native β cells. It has been estimated that fewer than 40% of patients are able to reach and maintain a euglycaemic state over a life-long insulin regimen (4).

Therefore, while insulin therapy can maintain acceptable glycemic levels and reduce diabetes-related complications, it is not a cure: the only real way to definitively treat diabetes is to restore the beta cell mass or the lost functionality of those cells.

Whole-pancreas transplantation has become the gold standard treatment to restore durable glycemic control and improve patient survival. However, as it is a major surgical intervention and requires life-long immunosuppression, this procedure is only proposed to selected patients (5), and is severely limited by organ shortage (6).

Replacement therapy using cadaveric islet transplantation has been proposed since the 1970s (7). The results of islet transplantation, initially providing an insulin-independence rate lower than 20% after 1 year, noticeably improved with the introduction of the Edmonton protocol (8), achieving glycaemia stabilization in 88% of patients after 1 year and 71% after 2 years (9).

Islet transplantation is performed with a transhepatic portal infusion, does not require a surgical procedure, and carries low morbidity. The major complications following islet transplantations are portal vein thrombosis and bleeding, for which emergency laparotomies are rarely necessary (10). For those reasons, islet transplantation is preferred to solid pancreas transplantation in fragile patients. In contrast, even there are no direct, randomized trials comparing the outcomes, the results in term of insulin independence are slightly inferior compared to whole pancreas transplantation (11).

In the recent past, the use of stem cells for T1D has expanded enormously, shifting from adult stem cells (mostly represented by bone-marrow derived hematopoietic and mesenchymal stem cells) to pluripotent stem cells (12). Previously, progress made possible the differentiation of embryonic stem cell populations (ESCs) into functional β cell clusters, providing a source of islets suitable for replacement therapy (13).

Regenerative medicine and tissue/organ engineering aim to improve the length and quality of patients' life by regenerating, preserving or enhancing the original tissue/organ function. In this context, a variety of novel methods have been considered to address tissue/organ insufficiency, including stem cell-based therapies for the regeneration of damaged tissues and tissue/organ-engineered organs to replace tissue/organ function.

Additionally, a complementary work stream, known as cell-on-scaffold technology, aims at creating the “ideal” biological template to be repopulated with specific cells in order to obtain a functional bio-engineered pancreas (14).

The aim of this document is to give an overview of the existing knowledge of current experimental strategies in the treatment of diabetes covered by the umbrella of regenerative medicine.

## Stem cells and diabetes

Stem cell biology has offered fascinating solutions to restore the insufficient production of insulin resulting from the loss or dysfunction of pancreatic β-cells.

Theoretically, stem cells (embryonic stem cells—ESC) can differentiate into functional β-cell populations following specific pathways and migrate to the damaged tissue in order to guarantee an appropriate β-cell mass (15). Alternativley, stem cells can be induced to differentiate *in vitro* into insulin-producing cells (3).

For both *in-vivo* and *in-vitro* approaches, the most important problem is choosing the best type of progenitor cell.

The most investigated types of stem cells for diabetes treatment are:

- Embryonic stem cells (ESCs) (16–18)
- Germline stem cells (19, 20)
- Mesenchymal stem cells (MSCs) (21, 22–23)
- Induced pluripotent stem cells (iPSCs) (3, 24, 25).

Despite a significant effort to produce translational results from bench to bedside, there is currently no cure for diabetes, moreover, as reviewed by Lilly et al. (1) each of the four stem cell types present significant issues.

### Embryonic stem cells

Though promising, the use of embryonic stem cells involves ethical constraints and a high risk of the development of teratomas (26).

In 2000, Soria et al. successfully isolated pancreatic insulin-producing cells (IPCs) produced by the introduction the human insulin gene into mouse ESCs. Cells were then transplanted into the spleen of streptozotocin-induced diabetic animals, obtaining transient glycaemia normalization and body weight normalization within 1 week. Nevertheless, for unknown reasons, about 40% of ESCs-implanted mice became hyperglycemic again within 12 weeks after the implantation (13).

In 2005, another group explored the capability of insulin-producing cells to reverse hyperglycemia using a streptozotocin (STZ)-induced diabetic NOD/SCID mouse model. Clusters formed by GFP-labeled ES insulin-producing cells were transplanted into the kidney sub-capsular space of diabetic mice (each cluster contained 100 to 150 insulin-positive cells). Cellular transplantation reversed the hyperglycemic state for 3 weeks, but the rescue failed due to immature teratoma formation (27).

### Germline stem cells

Although pluripotent cells have been confirmed as a stem cell source using female germline stem cells, the production of functional β-cells still needs to be explored *in-vivo* (20).

### Mesenchymal stem cells

Thus far, MSC treatment has been used to address the autoreactive host immune system in T1D. T1D is an autoimmune disease in which insulin-producing pancreatic β cells are destroyed by the autoreactive host immune system. To definitively cure T1D, this autoreactive host immune system must be first addressed before any attempts are made at islet replacement or regeneration. The immunomodulatory effect of MSCs has been explored in an attempt to prevent immune diseases in the past decades, but several issues remain unsolved.

Mesenchymal stem cell therapy continues to be a “mild” tool and may not be an efficacious treatment to reverse autoimmunity of T1D without the co-administration of immunosuppressive drugs (still necessary to prevent the acute autoimmunity reaction). The effects are incomplete and provisional, requiring chronic administration or additional therapies (28).

Furthermore, MSCs need the guidance of “homing” factors to reach the desired sites of action, but most homing factors, especially the homing factors directed at the pancreas, are still unknown. Finally, MSCs injected intravenously suffer from a “pulmonary first pass effect” and are likely to be sequestered in the lungs (29).

### Induced pluripotent stem cells

The use of iPS cells may be a suitable treatment option, allowing the use of pluripotent cells without manipulating embryos and offering the possibility of generating patient-specific cells. Moreover, recent data by Russel et al. demostrated the possibility of using these cells to overcome not only the alloresponse but also the auto-immune reaction in Type 1 Diabetes. Indeed, genome editing of iPSC demonstrated the capability of available technology to generate invisible cells that are able to escape immune reactive cells (30). However, iPSCs have mutagenic potential in some reprogramming methods, and limitations for long-term transplant viability and functionality (31).

## β cells from direct reprogramming

The clinical application of stem cells for the cure of diabetes still has many roadblocks to overcome. For this reason, different groups of researchers have explored the direct reprogramming of non-β adult cells into insulin-producing cells in order to exploit the production of new bona fide β cells *in-vitro*.

This technology, known as transdifferentiation, is based on the misexpression of specific groups of master regulatory transcription factors able to control the transition from one progenitor cell state to the next, ultimately generating mature insulin-producing cells (32–34).

Specifically, this process has been applied to:

- Pancreatic endocrineαcells (35–37)
- Pancreatic exocrine acinar cells (38)
- Hepatic cells (39, 40).

### Pancreatic endocrine α cells

In order to investigate a new source of β-cells, the transdifferentiation of α-cells cells is attractive due to common endodermic origin of β- and α-cells.

In 2009, the research group led by Collombat demonstrated *in-vivo* the conversion of alpha cells into functional beta cells by the ectopic overexpression of the transcription factor Pax4 during the development, (35) or the loss of Aristaless-Related Homeobox (Arx) restoring euglicemia in STZ-induced diabetic mice (36).

At the molecular level, the β-cell factor Pax4 works by inhibiting the α-cell master regulatory transcription factor Arx. Therefore, the absence of Arx alone is sufficient to switch α-cells to β-cells.

### Pancreatic exocrine acinar cells

Transdifferentiation protocols can be also applied to the pancreatic exocrine cellular component, which amounts to ~98% of the whole adult pancreas.

Pancreatic acinar cells and pancreatic duct cells are the most represented exocrine cellular types.

It must also be highlighted how exocrine cells comprise the vast majority of cells discarded from all cadaveric donor pancreata during the traditional islet isolation process. If successful, reprogramming could take advantage of a large pool of cells for conversion to β cells that would otherwise not be used.

The overexpression of specific transcription factors such as insulin promoter factor 1 (PDX1), neurogenin-3 (NGN3), and musculoaponeurotic fibro sarcoma oncogene family A (MafA) have showed, after viral transfection, evidence of acinar to β conversion in Rag 1^−/−^ non-diabetic animals. This overexpression is not sufficient to reverse diabetes in STZ-induced mice, though it does partially correct the hyperglycemic state (38).

### Hepatic cells

Starting from the same embryonic origin as β-cells and sharing analogous glucose-sensing systems, hepatocytes have been targeted for transdifferentiation into pancreatic β-cells by genetic reprogramming (39).

This possibility was explored and validated for the first time in 2000 by Ferber S and colleagues who published the first reports of transdifferentiation of liver cells. This group produced mice with a recombinant adenovirus that induced the expression of endogenous PDX-1 and the expression of other β-cell markers and resulting in substantial insulin (both hepatic and plasma immunoreactive insulin) (40).

## Encapsulation technology

Two new promising insulin delivery technologies are under investigation: micro- and macro- encapsulation devices.

Regardless of the capsule size (micro- or macro-), this approach aims to wrap the islets in a biocompatible membrane that permits the diffusion of nutrients while sheilding the islets from larger molecules, including antibodies and immune cells.

Proposed by Via-Cyte^1^, a privately-held regenerative medicine company developing novel cell replacement, PEC-Direct™ and PEC-Encap™ (VC-01™) are the first and second generation β-cell-derived product delivering technologies.

PEC-Direct™ is a macrodevice designed to allow direct vascularization of specific pancreatic progenitor cells (referred as PEC-01™), guaranteeing their maturation and *in-vivo* differentiation into insulin-producing cells (41, 42).

Despite the potentially groundbreaking results, one of the major limitations of the product is the direct contact of the transplanted cells with the circulating cells of the host, implying the need for immunosuppressive therapy.

PEC-Encap™ (VC-01™) aims to overcome this problem by incorporating Encaptra™ technology, a fine permeable film that permits surface vascularization and diffusion across the membrane in order to avoid contact between the transplanted and host cells. This improvement supplies an immunological protection that theoretically avoids the requirement of life-long immunosuppression.

ViaCyte received the approval from the U.S. Food and Drug Administration (FDA) in August 2014 to begin evaluation in human clinical trials. The PEC-Encap clinical trial (STEP ONE trial) is currently evaluating basic safety and tolerability in patients with type 1 diabetes in Canada and the USA.

On August 1st 2017, the company presented the first patient implanted with PEC-Direct™.

## Engineering a 3-D niche for islets: synthetic and prefabricated scaffolds

In their original milieu, islets are surrounded by pancreas-specific ECM proteins, usually composed of interstitial matrix and basement membrane proteins such as collagen type IV, laminin, and fibronectin. During isolation, islets are deprived of this matrix, which results in a loss of graft funtion (43) due to the crucial role that the matrix plays in islet survival, function, and proliferation (44).

The idea to house islets within a synthetic biomaterial at alternate transplantation sites is a potential therapeutic option. This concept is based on the goal of engineering a three-dimensional platform able to provide a non-toxic environment for seeded cells and recreate the native physiological milieu.

Different fundamental requisites for cellular transplantation have been identified including porosity, biocompatibility, the ratio between surface area and volume, and a suitable environment for new tissue formation that can integrate with the surrounding tissue (45).

The porosity of the scaffold is considered to be a crucial property for cellular vitality, guarantying the effective delivery of oxygen and nutrients to cells. Micro- and macroporous synthetic scaffolds are characterized by pores with a diameter under or over 50 μm respectively.

This approach has numerous advantages: the choice of biomaterials to use is wide, many biomaterials provide a relatively precice and repetitive assembly at the micrometer level, and the material used can be loaded or cross-linked with numerous molecules in the attempt to augment cellular functionality (46, 47).

Important synthetic polymers that have been investigated are chitosan (48), polylactic-co-glycolic acid (PLGA) (49, 50) and poly-L-lactide acid (PLA) (46).

In 2017, Smink et al. explored a scaffold composed by polyD,L-lactide-co-e-caprolactone (PDLLCL), commercially available as Neurolac [http://polyganics.com/products].

The group tested other polymers (polyethylene oxide terephthalate)/polybutylene terephthalate (PEOT/PBT) and polysulfone), but concluded that the PDLLCL-based scaffold was the only polymer that supported *in-vitro* rat islet survival. After the *in-vitro* testing, islets seeded in a PDLLCL scaffold were finally transplanted into a diabetic rat model resulting in normoglycemia within 3 days and for the duration of the 16 week study period (47).

A macroporous scaffold made by poly(dimethylsiloxane) (PDMS) has been explored by Pedraza et al. (51). PDMS scaffolds were prepared by using the solvent casting and the particulate leaching procedure and were pre-conditioned for islet loading via washing with islet culture media. For *in-vitro* investigations, each scaffold was loaded with 1,500 rat or human islet equivalents (IEQ).

When seeded on PDMS-based scaffold, islets showed an *in-vitro* enhanced viability compared to 2D culture controls under low oxygen tensions. The *in-vivo* effectiveness of scaffolds to support rat islet grafts was assessed after transplantation in the omental pouch of streptozotocin-induced diabetic syngeneic rats (in this case 1,800 IEQ were used) achieving normoglycemia with a mean reversal time of 1.8 ± 1.3 days.

These data have paved the way for the use of a pre-fabricated scaffold transplanted in the omental pouch in clinical practice. In 2017, Baidal described the use of a biological scaffold seeded by autologous islets to treat a 43-year-old patient with a 25-year history of type 1 diabetes. The authors reported a laparoscopic islet transplantation (for a total of 602,395 IEQ) onto the greater omentum in a degradable biologic scaffold composed by alternate layers of islets, recombinant thrombin (Recothrom) and autologous plasma. In this patient, euglicemia was restored and insulin independence achieved, but data from long-term follow-up are still missing (52).

Although these results are promising, several issues should be still addressed. As suggested by Pellicciaro et al. (53), we need to know how long transplanted islets remain in hypoxic conditions, and how rapidly islets are re-vascularized. This question is still unanswered due to the fact that the oxygenation conditions of transplanted islets in the omentum are still debated.

## Extracellular matrix scaffold and pancreas bioengineering for diabetes treatment—tissue engineering and regenerative medicine TE/RM approach

In contrast with solid pancreas transplantation, treatment with islets frequently requires multiple injections to reach the minimal functional mass able to grant insulin independence. Furthermore, the long-term exhaustion of the transplanted islets often requires additional transplantations in order to maintain the results. Therefore, multiple pancreas donations often go to one single patient, making the treatment extremely resource-consuming (54).

Host instant blood mediated inflammatory reaction (IBMIR) (55, 56), lack of revascularization of the site of implantation in the early post-transplant phase (57) and recurrence of autoimmunity (58) are common examples of the many other hurdles affecting the outcome of islet transplantation.

As discussed above, encapsulation has been developed to overcome some of these issues. However, the complete isolation of the islets from the surrounding tissue is deleterious because it does not allow for normal exchange with the surrounding milieu. The extreme effect of this isolation is “anoikis,” defined as programmed cellular apoptosis induced by inadequate or inappropriate cell-matrix interactions (59, 60).

In this scenario, the “ideal” islet transplantation procedure would also provide an extracellular matrix environment that promotes isolated β-cell mass survival and function (61, 62).

Due to the importance of the three-dimensional extracellular matrix (ECM), a relatively new technology is under evaluation to produce acellular ECM-based scaffolds that can be repopulated with patients' autologous cells.

The cell-on-scaffold technology stems from the ability to strip the cellular component from a tissue or a solid organ using a technique known as decellularization. The result is a three-dimensional acellular template composed only by the ECM. This structure maintains the original macro- and micro- architecture of the organ as well as all the biochemical cues of the ECM itself (63).

The goal is to produce an endless source of transplantable organs through the repopulation (recellularization) of biological scaffolds of animal origin, giving rise to a totally new era in the field of transplantation (64).

This approach has been successfully applied in experimental models involving insulin-producing cells. De Carlo et al. proposed one of the first experimental models in 2010 (65), seeding an acellular matrix of pancreatic and hepatic scaffolds with rat islets. The final construct was implanted in streptozotocin-induced diabetic rats that showed reduced exogenous insulin requirements.

In a mouse model, Goh et al. suggested a perfusion-based decellularization protocol able to produce an acellular pancreas-specific template (66). The whole pancreas was harvested and perfused through the portal vein with a specific detergent to completely remove the parenchymal cellular counterpart. For recellularization, AR42J (acinar cell line) and MIN-6 β-cells were selected. Acinar cells were seeded directly into the pancreas by retrograde perfusion through the pancreatic duct, whereas MIN-6 β-cells were introduced through the hepatic vein via a multistep injection. The obtained construct was then maintained in static culture for 5 days. The recellularization showed the engraftment of both cell types with an apoptosis rate of 18% and the preservation of insulin expression.

More recently, this protocol has been optimized comparing different strategies for perfusion-based decellularization procedures (67) in a rat model. Three different perfusion routes (arterial, venous, and pancreatic) were tested for decellularization as well as for repopulation with islets. Although no significant differences were observed between the groups in the obtainment of the acellular scaffold, the pancreatic duct was shown to be the best way to repopulate the scaffold, as it avoided extra-parenchymal leakages and showed an 80% seeding efficiency.

In order to achieve clinically relevant sized scaffolds, porcine models were investigated as a platform for pancreas bioengineering (68).

The decellularization protocol involved the perfusion (0.75 L/h) of a single detergent (1% Triton X-100) through the pancreatic duct for 12 h. After a final rinse with sterile PBS, the scaffold was seeded with 2 different cell types:

- human amniotic fluid-derived stem cells (hAFSC), to assess the cellular compatibility of the acellular pancreas ECM.
- porcine islets, to demonstrate the potential of the ECM to support pancreatic function.

Finally, by using a metabolic assay, an increase in the metabolic rate of seeded islets was observed between day 3 and 7, with insulin secretion compared to isolated non-seeded islets. Furthermore, it was noticed that after 72 h, islet insulin production was pulsatile under basal and high glucose conditions.

This research corroborated the theory that a porcine pancreas can serve as a platform for insulin-producing bioengineered tissue. Porcine pancreata can be harvested and decellularized while retaining the native architectural and biochemical cues. The resultant ECM can be seeded with the critical mass of islets required to meet insulin requirements.

The temperature of the detergent plays a central role in the preparation of the scaffold and notably on the biological quality of the final ECM. As described by Sumitran-Holgersson et al. (69), a three-step infusion of 4°C sodium deoxicholate, Triton X-100 and DNase for the production of an acellular porcine scaffold was more effective than normo-thermic decellularization. Interestingly, these scaffolds were seeded with human fetal pancreatic stem cells (hFPSCs), supporting both the endocrine and exocrine functions under static culture conditions. The endocrine properties of the seeded cells were evaluated by the synthesis of C-peptide, glucagon and the expression of PDX1, whereas the tissutal exocrine capacity was assessed by α-amylase secretion levels.

Recently, human pancreata discarded for clinical transplantation were used to produce acellular, extracellular matrix-based scaffolds (70). This study successfully demonstrated that that a whole human pancreas could be decellularized through a detergent-based perfusion protocol with the clearance of cells and HLA class I and II antigens. The scaffolds obtained this way showed the preservation of the ECM architecture (at all hierarchical levels), as well as composition and mechanical properties. In addition, this study showed that cell proliferation, glucose metabolism, and several growth factors (key players in essential pathways such as angiogenesis) are retained within the 3D structure of acellular pancreatic matrix. Finally, the authors showed that the pancreatic scaffold inhibits naïve CD4+ cell proliferation, promotes their apoptosis, and induces their conversion into T regulatory cells (T-regs), suggesting immunomodulating properties of the model.

As a corollary, if successful, the use of human pancreata as source to produce acellular scaffolds could provide an allogeneic platform on which to repopulate endocrine cell populations. In terms of numbers, this alternative to animal sources would allow for the use of 30% of discarded pancreata that were originally harvested for transplantation purposes but finally discarded in the US (71, 72).

The ECM can also be incorporated in multicellular spheroids together with different cell typologies enhancing their regenerative properties. Human amniotic epithelial cells (hAECc) obtained from placental samples are broadly accessible, and have immunomodulatory, anti-inflammatory and regenerative abilities (73, 74). In their preliminary data, Lebreton et al. produced heterospheroids consisting of rat islets and hAECc (with a ratio of 1:1) that were ultimately implanted under the kidney capsule of diabetic severe combined immunodeficiency (SCID) mice. Blood glucose tests and IPGTTs revealed an enhanced glycemic control compared with control animals. In this condition, the cumulative percentage of animals reaching normoglycemia was 74% in the islet + hAECc group vs. 26% in the islets-alone group. Even if preliminary, these data suggest that hAECs could have a meaningful potential to protect islet cells and they could be utilized to improve islet cell survival and function prior to transplantation when incorporated in heterosphere.

According to the classical definition for the “ideal biomaterial,” it would have adequate biomechanical properties, a low risk for infection transmission disease and would not elicit an unfavorable immune response (75). Theoretically, an ECM-based scaffold could be considered particularly suitable but its immunogenicity has to be considered one of the major drawbacks.

Based on a plethora of factors and parameters including graft complexity or the amount of specific protein families, the whole graft can generally be considered immunogenic or antigenic, and the ECM is no exception.

This point has been highlighted and discussed in 2006 (76). The author reviewed the common use of porcine and bovine biomaterials (e.g., Veritass®, CuffPatch™, TissueMends®) as clinical examples of acellular non-autologous biological structures without reporting any adverse immunological reactions. In this context, he also reported numerous studies regarding the presence of small amounts if gal-epitope (galactosyl 1,3 galactose epitope) following engraftment, which were unable to induce the complement activation or a cell-mediated rejection (77, 78). The gal-epitope has been deeply studied in this field, and non-gal-epitopes (such as α-enolase or E-cadherin) also seem to be actively involved in the immune reaction (79) to xenogeneic grafts.

As suggested by Wiles et al. (80), the decellularization process—by removing the antigens responsible for an immune response—could minimize both acute and chronic rejection. However, decellularized tissue may still provoke an immune response process by the presence of damage-associated molecular pattern proteins (DAMPs) (81). DAMPs are well represented not only in the native cellular tissue, but also in the acellular scaffold actively secreted during cellular injuries and necrosis. Their presence upregulates HMGB1, (High mobility group box 1) a highly abundant, ubiquitous protein that can promote the pathogenesis of inflammatory and autoimmune diseases once it is in an extracellular location (82).

Immunoisolation could be a solution to isolate the xenogeneic scaffold from the surrounding environment. Immunocloaking has provided us with important preliminary data by perfusing the organ with a nanofilm (called ImmunoCloak) made by subendothelial ECM able to camouflage the antigenic components. This nanobarrier guarantees the permanent exchange of oxygen and metabolites between the systemic bloodstream and the transplanted organ while masking antigens. Using this solution, Brasile et al. delayed the onset of rejection significantly, from 6 days for untreated kidneys up to 30 days for treated “ImmunoCloaked” kidneys. (83). Even with significant limitations (above all the fact that the membrane degrades after 1 month), it is reasonable that this technology could be used in order to mask the residual acellular scaffold immunogenity (84).

## Extracellular matrix and stem cells as potential combination

Cell-matrix interactions have been proven to be essential not only for cellular proliferation, but also for differentiation (85) and physiological function (86). Based on this evidence, the idea is to use the organ-specific environment—created by the ECM—in order to offer the implanted cells the best growing conditions in order to obtain (*in-vitro)* a transplantable organoid.

Specifically, the relationship between the ECM and islets has been deeply investigated, even if the understanding of the exact role of extracellular matrix in the functional endocrine system remains unclear.

It has been shown that the ECM plays a pivotal role in the formation of the correct stem cell niche by specific sequences of interplays (87).

Recently, an international Lancet commission has evaluated the use of stem cells, underlining the most important hurdles that are currently in the spotlight (88).

They redefined the use of stem cells both for TE/RM as well as for cellular therapy, highlighting the lack of understanding and the importance of having a solid scientific consciousness, that in some cases has failed to fulfill the original promise: help the patient (89).

## Conclusions

The pancreatic endocrine component is an interesting arena for regenerative medicine and cell therapy. Although in its early days, the evolution of TE/RM and the study of stem cell biology is leading to innovative treatments in the therapeutic field.

In this text, we reviewed studies reporting successful strategies for pancreatic tissue engineering, which are based on stem cells, islet encapsulation, and scaffold technologies. Fueled by the encouraging results, we propose that the combination of TE/RM and the stem cell approach could lead to the creation of a bioartificial pancreas. Although still far from becoming a clinical reality, the potential application of this futuristic approach is almost unlimited.

## Author contributions

AP, AC conceived, designed and wrote the manuscript. TZ, LC, and CMB participated in the review draft design, provided the appropriate literature, revised it critically and approved the final version. AK-Q revised the English, revised critically the manuscript and approved the final version. EB wrote the paragraph regarding the encapsulation technology, revised critically the manuscript and approved the final version. AA, LP, TB, CT and GO conceived, designed and supervised the whole manuscript, revised it critically and approved the final version.

### Conflict of interest statement

The authors declare that the research was conducted in the absence of any commercial or financial relationships that could be construed as a potential conflict of interest.

## Footnotes

^1^http://viacyte.com

## References

[1] Lilly MA Davis MF Fabie JE Terhune EB Gallicano GI. Current stem cell based therapies in diabetes. Am J Stem Cells (2016) 5:87–98. 27853630 PMC5107653

[2] American Diabetes Association Economic costs of diabetes in the U.S. in 2012. Diabetes Care (2013) 36:1033–46. 10.2337/dc12-2625 23468086 PMC3609540

[3] Pagliuca FW Millman JR Gurtker M Segel M Van Dervort A Ryu JH. Generation of functional human pancreatic β cells *in vitro*. Cell (2014) 159:428–39. 10.1016/j.cell.2014.09.040 25303535 PMC4617632

[4] Orlando G Stratta RJ Light J. Pancreas transplantation for type 2 diabetes mellitus. Curr Opin Organ Transplant. (2011) 16:110–5. 10.1097/MOT.0b013e3283424d1f 21150617

[5] Lombardo C Perrone VG Amorese G Baronti W Marchetti P Boggi U. Update on pancreatic transplantation on the management of diabetes. Minerva Med. (2017) 108:405–18. 10.23736/S0026-4806.17.05224-7 28466634

[6] Kandaswarmy R Stock PG Gustafson SK Skeans MA Curry MA Prentice MA OPTN/SRTR 2015 annual data report: pancreas. Am J Transplant. (2017) 17:117–73. 10.1111/ajt.14125 28052606

[7] Sutherland DE Matas AJ Najarian JS. Pancreas and islet transplantation. World J Surg. (1977) 2:185–95. 10.1007/BF01665079 405802

[8] Shapiro AM Lakey JR Ryan EA Korbutt GS Toth E Warnock GL. Islet transplantation in seven patients with type 1 diabetes mellitus using a glucocorticoid-free immunosuppressive regimen. N Engl J Med. (2000) 343:230–8. 10.1056/NEJM200007273430401 10911004

[9] Hering BJ Clarke WR Bridges ND Eggerman TL Alejandro R Bellin MD. Phase 3 trial of transplantation of human islets in type 1 diabetes complicated by severe hypoglycemia. Diabetes Care (2016) 39:1230–40. 10.2337/dc15-1988 27208344 PMC5317236

[10] Ahearn AJ Parekh JR Posselt AM. Islet transplantation for Type 1 diabetes: where we are now? Expert Rev Clin Immunol. (2015) 11:59–68. 10.1586/1744666X.2015.978291 25454816

[11] Barton FB Rickels MR Alejandro R Hering BJ Wease S Naziruddin B. Improvement in outcomes of clinical islet transplantation: 1999-2010. Diabetes Care (2012) 35:1436–45. 10.2337/dc12-0063 22723582 PMC3379615

[12] Skyler JS. Hope vs. hype: where are we in type 1 diabetes? Diabetologia (2018) 61:509–16. 10.1007/s00125-017-4530-x 29275427

[13] Soria B Roche E Bernà G Leon-Quinto T Reig JA Martín F. Insulin-secreting cells derived from embryonic stem cells normalize glycemia in streptozotocin-induced diabetic mice. Diabetes (2000) 49:157–62. 10.2337/diabetes.49.2.157 10868930

[14] Salvatori M Katari R Patel T Peloso A Mugweru J Owusu K. Extracellular matrix scaffold technology for bioartificial pancreas engineering: state of the art and future challenges. J Diabetes Sci Technol. (2014) 8:159–69. 10.1177/1932296813519558 24876552 PMC4454093

[15] Guo T Hebrok M. Stem cells to pancreatic β-cells: new sources for diabetes cell therapy. Endocr Rev. (2009) 30:214–27. 10.1210/er.2009-0004 19389995 PMC2726841

[16] Kroon E Martinson LA Kadoya K Bang AG Kelly OG Eliazer S. Pancreatic endoderm derived from human embryonic stem cells generates glucose-responsive insulin-secreting cells *in vivo*. Nat Biotechnol. (2008) 26:443–52. 10.1038/nbt1393 18288110

[17] D'Amour KA Bang AG Eliazer S Kelly OG Agulnick AD Smart NG. Production of pancreatic hormone-expressing endocrine cells from human embryonic stem cells. Nat Biotechnol. (2006) 24:1392–1401. 10.1038/nbt1259 17053790

[18] Jiang J Au M Lu K Eshpeter A Korbut G Fisk G. Generation of insulin-producing islet-like clusters from human embryonic stem cells. Stem Cells (2007) 25:1940–53. 10.1634/stemcells.2006-0761 17510217

[19] Noggle S Fung HL Gore A Martinez H Satriani KC Prosser R. Human oocytes reprogram somatic cells to a pluripotent state. Nature (2011) 478:70–5. 10.1038/nature10397 21979046

[20] Yamada M Johannesson B Sagi I Burnett LC Kort DH Prosser RW. Human oocytes reprogram adult somatic nuclei of a type 1 diabetic to diploid pluripotent stem cells. Nature (2014) 510:533–6. 10.1038/nature13287 24776804

[21] Bassi EJ Moraes-Vieira PM Moreira-Sa CS Almeida DC Vieira LM Cunha CS. Immune regulatory properties of allogeneic adipose-derived mesemchymal stem cells in the treatment of experimental autoimmune diabetes. Diabetes (2012) 61:2534–45. 10.2337/db11-0844 22688334 PMC3447906

[22] Gao X Song L Shen K Wang H Qian M Niu W. Bone marrow mesenchymal stem cells promote the repair of islets from diabetic mice through paracrine actions. Mol Cell Endocrinol. (2014) 388:41–50. 10.1016/j.mce.2014.03.004 24667703

[23] Hao H Liu J Shen J Zhao Y Liu H Hou Q. Multiple intravenous infusions of bone marrow mesemchymal stem cells reverse hyperglycemia in experimental type 2 diabetes rats. Biochem Biophys Res Commun. (2013) 436:418–23. 10.1016/j.bbrc.2013.05.117 23770360

[24] Hrvatin S O'Donnel CW Deng F Millman JR Pagliuca FW Dilorio P Differentiated human stem cells resemble fetal, not adult, beta cells. Proc Natl Acad Sci USA. (2014) 111:3038–43. 10.1073/pnas.1400709111 24516164 PMC3939927

[25] Nair G Hebrok M Islets formation in mice and men: lessons for the generation of functional insulin-producing β-cells from human pluripotent stem cells. Curr Opin Genet Dev. (2015) 32:171–80. 10.1016/j.gde.2015.03.004 25909383 PMC4523641

[26] Naujok O Francini F Picton S Bailey CJ Lenzen S Jörns A. Changes in gene expression and morphology of mouse embryonic stem cells on differentiation into insulin-producing cells *in vitro* and *in vivo*. Diabetes Metab Res Rev. (2009) 25:464–76. 10.1002/dmrr.965 19425055

[27] Fujikawa T Oh SH Pi L Hatch HM Shupe T Peteresen BE Teratoma formation leads to failure of treatement for type 1 diabetes using embryonic stem cell-derived insulin-producing cells. Am J Pathol. (2005) 166:1781–91. 10.1016/S0002-9440(10)62488-1 15920163 PMC1602425

[28] Wu H Mahato RI. Mesenchymal stem cell-based therapy for type 1 diabetes. Discover Med. (2014) 17:139–43. Available online at: http://www.discoverymedicine.com/Hao-Wu/2014/03/07/mesenchymal-stem-cell-based-therapy-for-type-1-diabetes/24641956

[29] Fisher UM Harting MT Jimenez F Monzon-Posadas WO Xue H Savitz SI Pulmonary passage is a major obstacle for intravenous stem cell delivery: the pulmonary first-pass effect. Stem Cells Dev. (2009) 18:683–92. 10.1089/scd.2008.0253 19099374 PMC3190292

[30] Gornalusse GG Hirata RK Funk SE Riolobos L Lopes VS Manske G. HLA-E-expressing pluripotent stem cells escape allogeneic responses and lysis by NK cells. Nat Biotechnol. (2017) 35:765–72. 10.1038/nbt.3860 28504668 PMC5548598

[31] Thatava T Nelson TJ Edukulla R Sakuma T Ohmine S Tonne JM. Indolactam V/GLP-1-mediated differentiation of human iPS cells into glucose-responsive insulin-secreting progeny. Gene Ther. (2011) 18:283–93. 10.1038/gt.2010.145 21048796 PMC3060028

[32] Cito M Pellegrini S Piemonti L Sordi V. The potential and challenges of alternative sources of βcells for the cure of type 1 diabetes. Endocr Connect. (2018) 7:R114–25. 10.1530/EC-18-0012 29555660 PMC5861368

[33] Sisakhtnezhad S Matin MM. Transdifferentiation: a cell and molecular reprogramming process. Cell Tissue Res. (2012) 348:379–96. 10.1007/s00441-012-1403-y 22526624

[34] Lysy PA Weir GC Bonner-Weir S. Making βcells from adult cells within the pancreas. Curr Diabetes Rep. (2013) 13:695–703. 10.1007/s11892-013-0400-1 23925431 PMC3798031

[35] Collombat P Xu X Ravassard P Sosa-Pineda B Dussaud S Billestrup N. The ectopic expression of Pax4 in the mouse pancreas converts progenitor cells into alpha and subsequently beta cells. Cell (2009) 138:449–62. 10.1016/j.cell.2009.05.035 19665969 PMC2792203

[36] Courtney M Gjernes E Druelle N Ravaud C Vieira A Ben-Othman N. The inactivation of Arx in pancreatic α-cells triggers their neogenesis and convertion into functional β-like cells. PLoS Genet. (2013) 9:e1003934. 10.1371/journal.pgen.1003934 24204325 PMC3814322

[37] Thorel F Nèpote V Avril I Kohno K Desgraz R Chera S. Conversion of adult pancreatic alpha-cells to beta-cells after extreme beta-cell loss. Nature (2010) 464:1149–54. 10.1038/nature08894 20364121 PMC2877635

[38] Zhou Q Brown J Kanarek A Rajagopal J Melton DA. *In vivo* reprogramming of adult pancreatic exocrine cells to β-cells. Nature (2008) 455:627–32. 10.1038/nature07314 18754011 PMC9011918

[39] Yang LJ. Liver stem cell-derived beta-cell surrogates for treatment of type 1 diabetes. Autoimmun Rev. (2006) 5:409–13. 10.1016/j.autrev.2005.10.009 16890895 PMC3414536

[40] Ferber S Halkin A Cohen H Ber I Einav Y Goldberg I. Pancreatic and duodenal homeobox gene 1 induces expression of insulin genes in liver and ameliorates streptozotocin-induced hyperglycemia. Nat Med. (2000) 6:568–72. 10.1038/75050 10802714

[41] Bruni A Gala-Lopez B Pepper AR Abualhassan NS Shapiro J. Islets cell transplantation of type 1 diabetes: recent advances and future challenges. Diabetes Metab Dyndr Obes. (2014) 7:211–23. 10.2147/DMSO.S50789 25018643 PMC4075233

[42] Kelly OG Chan MY Martison LA Kadoya K Ostertag TM Ross KG. Cell-surface markers for the isolation of pancreatic cell types derived from human embryonic stem cells. Nat Biotechnol. (2011) 29:750–6. 10.1038/nbt.1931 21804561

[43] Pileggi A Ricordi C Alessiani M Inverardi L Factors influencing Islets od Langerhans graft function and monitoring. Clin Chim Acta (2001) 310:3–16. 10.1016/S0009-8981(01)00503-4 11485749

[44] Coronel MM Stabler CL. Engineering a local microenvironment for pancreatic islet replacement. Curr Opin Biotechnol. (2013) 24:900–8. 10.1016/j.copbio.2013.05.004 23769320 PMC3783544

[45] Putnam AJ Mooney DJ. Tissue engineering using synthetic extracellular matrices. Nat Med. (1996) 2:824–6. 10.1038/nm0796-824 8673932

[46] Daoud JT Petropavlovskaia MS Patapas JM Degrandpre E Diraddo RW Rosenberg L. Long-term *in vitro* human pancreatic islet culture using three-dimensional microfabricated scaffolds. Biomaterials (2011) 32:1536–42. 10.1016/j.biomaterials.2010.10.036 21093908

[47] Smink AM Hertsig DT Schwab L van Apeldoorn AA de Koning E Faas MM A retrievable, efficacious polymer scaffold for subcutaneous transplantation of rat pancreatic islets. Ann Surg. (2017) 266:149–57. 10.1097/SLA.0000000000001919 27429018

[48] Elcin YM Elcin AE Bretzel RG Linn T. Pancreatic islet culture and transplantation using chitosan and PLGA scaffolds. Adv Exp Med Biol. (2003) 534:255–64. 10.1007/978-1-4615-0063-6_19 12903725

[49] Salvay DM Rives CB Zhang X Chen F Kaufman DB Lowe WL. Extracellular matrix protein-coated scaffolds promote the reversal of diabetes after extrahepatic islet transplantation. Transplantation (2008) 85:1456–64. 10.1097/TP.0b013e31816fc0ea 18497687 PMC2597660

[50] Gibly RF Zhang X Lowe WL Shea LD. Porous scaffolds support extrahepatic human islet transplantation, engraftment and function in mice. Cell Transplant. (2013) 22:811–9. 10.3727/096368912X636966 22507300 PMC3701739

[51] Pedraza E Brady AC Fraker CA Molano RD Sukert S Berman DM. Macroporous three-dimensional PDMS scaffolds for extrahepatic islet transplantation. Cell Transplant. (2013) 22:1123–35. 10.3727/096368912X657440 23031502 PMC4429907

[52] Baidal DA Ricordi C Berman DM Alvarez A Padilla N Ciancio G. Bioengineering of an intraabdominal endocrine pancreas. N Engl J Med. (2017) 376:1887–89. 10.1056/NEJMc1613959 28489987 PMC5572072

[53] Pellicciaro M Vella I Lanzoni G Tisone G Ricordi C The greater omentum as a site for pancreatic islet transplantation. CellR4 (2017) 5:e2410. PMC8025931 33834082

[54] Sordi V Pellegrini S Krampera M Marchetti P Pessina A Ciardelli G. Stem cells to restore insulin production and cure diabetes. Nutr Metab Cardiovasc Dis. (2017) 27:583–600. 10.1016/j.numecd.2017.02.004 28545927

[55] Agulnick AD Ambruzs DM Moorman MA Bhoumik A Cesario RM Payne JK Insulin-producing endocrine cells differentiated *in vitro* from human embryonic stem cells functions in macroencapsulation devices *in vivo*. Stem Cell Transl Med. (2015) 4:1214–22. 10.5966/sctm.2015-0079 PMC4572906 26304037

[56] Shimizu H Ohashi K Utoh R Isa K Gotoh M Yamato M. Bioengineering of a functional sheet of islet cells for the treatment of diabetes mellitus. Biomaterials (2009) 30:5943–49. 10.1016/j.biomaterials.2009.07.042 19674781

[57] Fotino N Fotino C Pileggi A. Re-engineering islet cell transplantation. Pharmacol Res. (2015) 98:76–85. 10.1016/j.phrs.2015.02.010 25814189 PMC4469528

[58] Pugliese A Reijonen HK Nepom J Burke GW III. Recurrence of autoimmunity in pancreas transplant patients: research update. Diabetes Manag. (2011) 1:229–38. 10.2217/dmt.10.21 21927622 PMC3171830

[59] Desai T Shea LD Advances in islets encapsulation technologies. Nat Rev Drug Discov. (2017) 16:338–50. 10.1038/nrd.2016.232 28008169 PMC11286215

[60] Frisch SM Screaton RA Anoikis mechanism. Curr Opin Cell Biol. (2001) 13:555–62. 10.1016/S0955-0674(00)00251-9 11544023

[61] Weber LM Hayda KN Anseth KS. Cell-matrix interactions improve beta-cell survival and isulin secretion in three-dimensional cuture. Tissue Eng Part A (2008) 14:1959–68. 10.1089/ten.tea.2007.0238 18724831 PMC2992396

[62] Stendahl JC Kaufman DB Stupp SI. Extracellular matrix in pancreatic islets: relevance to scaffold design and transplantation. Cell Transplant. (2009) 18:1–12. 10.3727/096368909788237195 19476204 PMC2724969

[63] Crapo PM Gilbert TW Badylak SF. An overview of tissue and whole organ decellularization processes. Biomaterials (2011) 32:3233–43. 10.1016/j.biomaterials.2011.01.057 21296410 PMC3084613

[64] Orlando G Soker S Stratta RJ. Organ bioengineering and regeneration as the new Holy Grail for organ transplantation. Ann Surg. (2013) 258:221–32. 10.1097/SLA.0b013e31829c79cf 23782908

[65] De Carlo E Baiguera S Conconi MT Vigolo S Grandi C Lora S. Pancreatic acellular matrix supports islet survival and function in a synthetic tubular device: *in vitro* and *in vivo* studies. Int J Mol Med. (2010) 25:195–202. 10.3892/ijmm_00000330 20043127

[66] Goh SK Bertera S Olsen P Candiello JE Halfter W Uechi G. Perfusion-decellularized pancreas as a natural 3D scaffold for pancreatic tissue and whole organ engineering. Biomaterials (2013) 34:6760–72. 10.1016/j.biomaterials.2013.05.066 23787110 PMC3748589

[67] Napierala H Hillebrandt KH Haep N Tang P Tintemann M Gassner J Engineer an endocrine Neo-Pancreas by repopulation of a decellularized rat pancreas with islets of Langerhans. Sci Rep. (2017) 7:41777 10.1038/srep41777 28150744 PMC5288794

[68] Mirmalek-Sani SH Orlando G McQuilling JP Pareta R Mack DL Salvatori M. Porcine pancreas extracellular matrix as a platform for endocrine pancreas bioengineering. Biomaterials (2013) 34:5488–95. 10.1016/j.biomaterials.2013.03.054 23583038 PMC3680884

[69] Elebring E Kuna VK Kvarnstrom N Sumitran-Hogelrsson S. Cold-perfusion decellularization of whole-organ porcine pancreas supports human fetal pancreatic cell attachment and expression of endocrine and exocrine markers. J Tissue Eng. (2017) 8:2041731417738145. 10.1177/2041731417738145 29118967 PMC5669317

[70] Peloso A Urbani L Cravedi P Katari R Maggsoudlou P Fallas ME. The human pancreas as a source of protolerogenic extracellular matrix scaffold for a new-generation bioartificial pancreas. Ann Surg. (2016) 264:169–79. 10.1097/SLA.0000000000001364 26649588 PMC4882269

[71] Kandaswamy R Skeans MA Gustafson SK Carrico RJ Tyler KH Israni AK. OPTN/SRTR 2013 Annual Data Report: pancreas. Am J Transplant. (2015) 15 (Suppl 2):1–20. 10.1111/ajt.13196 25626343

[72] Stratta RJ Gruessner AC Odorico JS Fridell JA Gruessner RW. Pancreas transplantation: an alarming crisis in confidence. Am J Transplant. (2016) 16:2556–62. 10.1111/ajt.13890 27232750

[73] Lebreton F Lavallard V Perez L Parnaud G Bosco D Berney T Islet Heterospheroids Generated From Islet Cells and Amniotic Epithelial Cells Reverse Diabetes After Marginal Mass Transplantation in a Murine Model. Available online at: https://confman.tts2018.org/mobis/lecture/674

[74] Lavallard V Lebreton F Perez L Parnaud G Bosco D Berney T Human Amniotic Epithelial Cells Integrated Into the Islet Heterospheroids Enhance Insulin Secretion and Protect Islet Cells From Hypoxic Injury. Available online at: https://confman.tts2018.org/mobis/lecture/920

[75] Mertsching H Schanz J Steger V Schandar M Schenk M Hansmann J. Generation and transplantation of bioartificial human tissue. Transplantation (2009) 88:203–10. 10.1097/TP.0b013e3181ac15e1 19623015

[76] Badylak SF The extracellular matrix as a biological scaffold material. Biomaterials (2007) 25:3587–93. 10.1016/j.biomaterials.2007.04.043 17524477

[77] McPherson TB Liang H Record RD Badylak SF. Galalpha(1,3)Gal epitope in porcine small intestinal submucosa. Tissue Eng. (2000) 6:233–9. 10.1089/10763270050044416 10941218

[78] Burlak C Wang ZY Chihara RK Lutz AJ Wang Y Estrada JL. Identification of human preformed antibody targets in GTKO pigs. Xenotransplantation (2012) 19:92–101. 10.1111/j.1399-3089.2012.00695.x 22497511

[79] Raeder RH Badylak SF Sheehan C Kallakury B Metzger DW. Natural anti-galactose alpha1,3 galactose antibodies delay, but do not prevent the acceptance of extracellular matrix xenografts. Transplant Immunol. (2002) 10:15–24. 10.1016/S0966-3274(01)00044-2 12182460

[80] Wiles K Fishman JM De Coppi P Birchall MA. The host Immune response to tissue-engineered organs: current problems and future directions. Tissue Eng Part B Rev. (2016) 22:208–19. 10.1089/ten.teb.2015.0376 26701069

[81] Daly KA Liu S Agrawal V Brown BN Johnson SA Medberry CJ Damage associated molecular patters within xenogeneic biologic scaffolds and their effects on host remodelling. Biomaterials (2012) 33:91–101. 10.1016/j.biomaterials.2011.09.040 21967802

[82] Magna M Pisetsky D. The role of HMGB1 in the pathogenesis of inflammatory and autoimmune diseases. Mol Med. (2014) 20:138–46. 10.2119/molmed.2013.00164 24531836 PMC3966993

[83] Brasile L Glowacki P Castracane J Stubenitsky BM. Pretransplant kidney-specific treatment to eliminate the need for systemic immunosuppression. Transplantation (2010) 90:1294–98. 10.1097/TP.0b013e3181ffba97 21076377 PMC6140766

[84] Orlando G. ImmunoCloak as a paradigm of the biomaterial approach to immunomodulation: where regenerative medicien meets organ transplantation. Transplantation (2017) 101:234–5. 10.1097/TP.0000000000001551 27798511

[85] Montesano R Mouron P Amherdt M Orci L Collagen matrix promotes reorganization of pancreatic endocrine cell monolayer into islet-like organoids. J Cell Biol. (1983) 97:935–9. 10.1083/jcb.97.3.935 6350323 PMC2112577

[86] Cheng JY Raghunath M Whitelock J Poole-Warren L. Matrix components and scaffolds for sustained islet function. Tissue Eng Part B Rev. (2011) 17:235–47. 10.1089/ten.teb.2011.0004 21476869

[87] Tuch BE Gao SY Lees JG Scaffolds for islets and stem cells differentiated into insulin-secreting cells. Front Biosci. (2014) 1:126–38. 10.2741/4199 24389176

[88] Cossu G Birchall M Brown T De Coppi P Culme-Seymour E Gibbon S. Lancet commission: stem cells and regenerative medicine. Lancet (2017) 391:883–910. 10.1016/S0140-6736(17)31366-1 28987452

[89] Abbott A Prestigious Karolinska Institute dismisses controversial trachea surgeon. Nat. News (2016). 10.1038/nature.2016.19629

